# Household Hints and Recipes

**Published:** 1879-01

**Authors:** 


### Household Hints & Recipes.

CHEAP PAINT.—Cheap paint for rough wood-work or fences is made of six pounds of melted pitch, one pint of linseed oil, and one pound of brick dust or yellow ochre. It is excellent, and will stand for years.

BRIGHT SCARLET INK.—The fiery scarlet ink so much used at present is simply a solution of eosine in water. Eosine is kept for sale by most wholesale druggists; the price is rather high, but the quantity needed is extremely small.

CEMENT FOR GLASS.—Dissolve 1 part of caoutchouc in 60 parts of chloroform; add 34 parts of mastic, and digest the whole for 8 days with a gentle heat. It is applied by means of a brush. This cement is particularly distinguished by its great transparency.

TO REMOVE WOOL FROM SHEEP SKINS, the flesh side is smeared with a mixture of lime and orpiment, and in twenty-four hours the hair comes off with the greatest ease. The proportion is about one pound of the sulphide of arsenic for six or eight pounds of quicklime.

HAIR CURLING LIQUID.—

Borax, 2 ounces.
Gum arabic, 2 drachms.
Spirit of camphor, 1 ounce.
Water, 2 pints.

On retiring moisten the hair with the liquid, and roll it into twists of paper in the usual manner.

MUSHROOM CATSUP.—Sprinkle the trimmed tops with salt, stir them occasionally for 2 or 3 days, then lightly press out the juice; add to each gallon of this ½ oz. each of bruised mustard seed and cloves, and 1 oz. each bruised allspice, black pepper, and gently simmer for an hour in a porcelain lined iron vessel; cool, strain, and bottle.

STARCH GLOSS.—This may be prepared as follows: Melt together 4 parts of pure stearin and 6 parts of paraffin, and pour the liquid into a cold form to solidify. Then cut the mass into pieces weighing about ¼ oz. Having made a lot of starch-paste, say from one pound of starch, one of these pieces is added and left in the mass during a few minutes' boiling. When using unboiled starch-paste, a small quantity of a hot solution of starch containing the gloss is applied to a rag, and the latter gently rubbed over the fabric just before ironing.

RED INK.—The following recipe for a beautiful red ink is given by Metra, of Paris: Dissolve 25 parts, by weight, of saffranine in 500 parts warm glycerine; then stir in carefully 500 parts alcohol and an equal quantity of acetic acid. It is then diluted with 9,000 parts water, in which is dissolved a little gum arabic.

PATENT-LEATHER VARNISH.—

Ivory-black, 6 parts.
Molasses, 2 parts.
Rape-oil, 1 part.
Sulphate of iron, 1 part.
Water, 8 parts.
Hydrochloric acid, comm., 1 part.
Sulphuric acid, comm., 1 part.

SAVING NOISE IN THE SICK-ROOM.—One of the most common annoyances to a feeble, nervous invalid is the noise attending the feeding of a coal fire. The harsh rattling of the coals grates upon the poor invalid's nerves most painfully. Here is an easy way to avoid it: Before bringing the coal into the room, divide it into little parcels, wrapping each in a piece of newspaper. These can be laid upon the fire, one at a time, without noise, thus saving the patient much inconvenience.

LIQUID BLACKING.—

Borax, 6 ounces.
Shellac, 1½ ounces.
Extract of logwood, 6 ounces.
Bichromate of potassa, 3 drachms.
Water, 2 gallons.

Dissolve the extract in one gallon of warm water, boil the shellac and the borax in a gallon of water till they are dissolved. Mix the two solutions together, and add the bichromate of potassa.

DIET.—J. S. Prescott calls attention to an experiment in dieting, which a person in Medina, Ohio, has practised with increasingly beneficial effects. As an economical experiment it certainly is forcibly interesting. We condense: "For breakfast, five Graham gems with butter; no inconvenience or hunger followed—cost *three cents.* Dinner, one-fourth pound of rice, one ounce each of sugar and butter—a good meal—cost *five cents.* Supper, one-fourth pound of corn meal, one-half pint of milk—cost *three cents.* One day's cost, eleven cents. For a change, one gill of beans which, by the quart, cost less than half a cent."

The correspondent claims to have worked hard, ate nothing between meals, is renewing his age and youthfulness, *and only dreads the lonesomeness to be experienced by living to a very great age.—N. Y. Shaker.*

INDELIBLE INK.—Indelible ink spots can be removed by using a solution of twenty grains of cyanide of potassium to half an ounce of water. Apply the solution to the article by means of a stiff camel's-hair brush. After the ink has been removed, wash the article thoroughly in water so as to get out all the cyanide. The utmost caution must be used in handling this cyanide of potassium, as it is a deadly poison. Care must be taken not to get any of the solution on a scratch or cut, as it is poisonous.

---

IMPROVED BLACK WRITING INK.—This ink is prepared by digesting for 24 hours, at a gentle heat, 7½ ounces of powdered nut-galls in a pint of alcohol of 82° per cent., and in another vessel, 2½ ounces of sulphate of iron, and the same quantity of gum arabic, with 3 pints of pure water. These two liquids, passed through a flannel strainer, are then to be mixed and allowed to settle for eight days, and again strained. The ink thus obtained does not form any deposit, nor does it mould, and is more permanent than any other.

---

CEMENT FOR MENDING RUBBER.—The best and simplest is probably a solution of virgin caoutchouc in bisulphide of carbon. The varnish, if any, should first be removed from the surface near the place to be mended. Then a piece of cloth of suitable shape and color is smeared on both sides with the cement, and applied with gentle pressure while the sides of the rent are kept close together. The rubber solution has the consistence of a stiff jelly, and, owing to the volatility of the menstrum, dries in a very short time.

---

ETCHING, ENGRAVING, OR WRITING UPON GLASS BY ELECTRICITY.—Cover the glass with a layer of solution of potassium nitrate, place into this a platinum wire connected with one of the poles of a battery. Then draw upon the glass, with the other electrode, which is also a platinum wire, the desired figures, letters or design. A plain mark will be left wherever the point of the electrode has been applied. Either electrode will answer; but the negative pole requires a less powerful battery than the other.

---

LEATHER-VARNISH.—The following formula is highly recommended by practical leather workers: Add to one pint of alcohol, in a bottle, 1¼ oz. of best shellac, ½ oz. of sandarac, and 80 grains of mastic, together with from 300 to 460 grains of Venice turpentine, to make it pliable and prevent it from cracking. The quantity of the latter depends on the nature of the leather, and must be determined experimentally. It is best to add a little coarse sand to the mixture, as a mechanical aid in breaking up the gums while shaking. Finally, a little aniline-black, dissolved in alcohol, is added, until the varnish has assumed a deep-black shade.

---

INDELIBLE INK.—

Nitrate of silver, 2 drachms.
Water, 6 drachms.
Mucilage, 1 drachm.
Ammonia, sufficient.

Dissolve the nitrate of silver in the water; add enough ammonia to redissolve the precipitate formed at first, and lastly the mucilage. Sometimes it is found convenient to color the ink with a little syrup of buckthorn, to facilitate the writing.

---

COUGH SYRUP.—One ounce of thoroughwort, one ounce of flaxseed; simmer together in one quart of water until the strength is entirely extracted; strain carefully; add one pint of best molasses and a half-pound loaf sugar; simmer them thoroughly together, and when cold bottle tight. A few doses of one teaspoonful at a time will alleviate the most distressing cough of the lungs, subdue any tendency to consumption, break up entirely the whooping cough, asthma, bronchitis, and all affections of the lungs and throat. It is simple, safe and effective.

---

CEMENT FOR AQUARIA, TANKS, ETC.—1. Equal parts of finely powdered pumice stone and powdered shellac—or, in place of the latter, sulphur—are fused together, and the mass is poured, while warm, into the joints. This mass will also cement together articles of glass, wood, or metal.

2. Equal parts of flowers of sulphur, powdered ammonium chloride, and finely sifted iron-filings are mixed with linseed-oil varnish, and enough barytes (barium sulphate) is added to make the mass firm.

---

HYGIENE OF BEDS.—Beds should be made of such material as will absorb as little as possible of dampness or impurities of any sort. One of the most important means of keeping a bed in a wholesome condition is thorough airing every morning. Immediately after rising, the occupant should throw open the bed, and the windows of the room, thus securing thorough removal of all the foul emanations from the body which accumulate during sleep. When this is not done, the foul products accumulate, and the bed may become a source of disease.

TO RESTORE FADED WRITING.—According to E. v. Bibra faded writing, originally written with nut-gall ink, may be restored by brushing over the paper, solution of ammonium sulphide, washing off the excess of the latter, and drying at a gentle heat or between folds of blotting paper. The intensity of the black marks, however, is not persisting. Or the writing may be brushed over with a concentrated solution of tannin, washing the excess off with water, at 60–70° C., and drying.

---

DAMP ROOMS.—Much sickness is the result of living in damp rooms. Brick houses, which are plastered directly upon the walls, are certain to be damp, as shown by the appearance of mold on the walls in various places, especially in dark closets, unventilated parlors, and spare bedrooms. A very good temporary remedy is to place in such rooms pans or other vessels containing freshly burned quicklime. It should be frequently renewed. Such rooms should be daily aired very thoroughly, and exposed to the rays of the sun. Mold should be removed from the wall as soon as it makes its appearance.

---

COFFEE AND MILK AS A REMEDY FOR CANINE DISTEMPER.—It will be interesting to lovers of the canine species to hear of a simple remedy for distemper. At the quarterly meeting of the Scottish Metropolitan Veterinary Medical Society, Mr. Baird mentioned the case of a colly dog in the last stage of the disease, and which its owner had determined to destroy. Shortly after being treated with doses of strong coffee and a little sweet milk, the animal, however, so far recovered as to be able to stand and walk. The chairman of the meeting said the case seemed almost unique.—*London Lancet.*

---

EBONY STAIN FOR WOOD.—Apple, pear, and walnut wood, especially of fine grain, give perfect imitations of ebony under the following treatment: Boil in a glazed vessel. with water, four ounces of gall-nuts, one ounce of logwood chips, half an ounce of vitriol, and half an ounce of crystallized verdigris; filter while warm, and brush the wood with the hot solution a number of times. The wood, thus stained black, is then to be coated two or three times (being allowed to dry completely after each coating) with a solution of one ounce of iron filings in a quart of good wine vinegar. This is to be prepared hot, and allowed to cool before use.

KEROSENE LINIMENT.—Dr. A. C. Hobbs, in the *Louisville Medical News*, recommends the following:

Kerosene oil, 2 ounces.
Tincture of opium, 4 drachms.
" " arnica, 5 "
" " stramonium, 4 drachms.
Aromatic spirit of ammonia, 6 drachms.
Spirit of camphor, 5 drachms.
Oil of origanum, 4 drachms.
Chloroform, 1 ounce.

Mix. An excellent liniment for sprains, bruises, soreness, and nervous pains; it is good for man and beast.

---

A NEW LIQUID GLUE.—To produce good glue size, dissolve in a copper pan, heated by indirect steam, 4½ to 5 lbs. of soda in 20 lbs. to 24 lbs. of boiling water; then add to it, stirring well at the time, 30 lbs. of powdered resin, keeping the whole continually boiling until all the resin is perfectly dissolved. This soda-resin composition, dissolved in the proportion of 1 lb. of resin to 30 lbs. to 40 lbs. of water, is to be mixed well together with a glue solution, made by dissolving 10 lbs. of glue in about 30 lbs. to 40 lbs. of water; then boil up both solutions together for about 10 minutes, after which run it through a fine sieve or filter, and it is then ready for use. The best proportions for mixing the vegetable and animal sizes are, for 1½ parts resin, add 11 parts glue.

---

MOUTH WASHES.—We find the following recommended in *Dental Science*, a periodical published in England:

WASH TO HARDEN THE GUMS.

Jamaica rum, 10 ounces.

Powdered alum, ½ drachm.

" saltpetre, ½ drachm.

" myrrh, 1 ounce.

A FAVORITE WASH.

Carbonate of potassa, 4 drachms.
Honey, 4 ounces.
Alcohol, 2 ounces.
Water, 10 ounces.
Oils of rose and wintergreen, sufficient.

FOR UNHEALTHY GUMS.

Carbolic acid, 20 drops.
Alcohol, 2 drachms.
Water, 6 ounces.

Use, first, a soft tooth brush with water, after which pour on a second tooth brush, slightly dampened, a little of the above lotion. After using this for a short time the gums become less tender, and the impurity of the breath, commonly caused by bad teeth, will be removed.

